# A Comparison of Closed Loop vs. Fixed Frequency tACS on Modulating Brain Oscillations and Visual Detection

**DOI:** 10.3389/fnhum.2021.661432

**Published:** 2021-06-23

**Authors:** Heiko I. Stecher, Annika Notbohm, Florian H. Kasten, Christoph S. Herrmann

**Affiliations:** ^1^Experimental Psychology Lab, Department of Psychology, European Medical School, Cluster of Excellence “Hearing4all”, Carl von Ossietzky University, Oldenburg, Germany; ^2^Department of Neurological Rehabilitation, Municipal Hospital of Bremen, Bremen, Germany; ^3^Research Center Neurosensory Science, Carl von Ossietzky University, Oldenburg, Germany

**Keywords:** transcranial alternating current stimulation, alpha, EEG, closed loop, visual perception

## Abstract

Transcranial alternating current stimulation has emerged as an effective tool for the exploration of brain oscillations. By applying a weak alternating current between electrodes placed on the scalp matched to the endogenous frequency, tACS enables the specific modulation of targeted brain oscillations This results in alterations in cognitive functions or persistent physiological changes. Most studies that utilize tACS determine a fixed stimulation frequency prior to the stimulation that is kept constant throughout the experiment. Yet it is known that brain rhythms can encounter shifts in their endogenous frequency. This could potentially move the ongoing brain oscillations into a frequency region where it is no longer affected by the stimulation, thereby decreasing or negating the effect of tACS. Such an effect of a mismatch between stimulation frequency and endogenous frequency on the outcome of stimulation has been shown before for the parietal alpha-activity. In this study, we employed an intermittent closed loop stimulation protocol, where the stimulation is divided into short epochs, between which an EEG is recorded and rapidly analyzed to determine a new stimulation frequency for the next stimulation epoch. This stimulation protocol was tested in a three-group study against a classical fixed stimulation protocol and a sham-treatment. We targeted the parietal alpha rhythm and hypothesized that this setup will ensure a constant close match between the frequencies of tACS and alpha activity. This closer match should lead to an increased modulation of detection of visual luminance changes depending on the phase of the tACS and an increased rise in alpha peak power post stimulation when compared to a protocol with fixed pre-determined stimulation frequency. Contrary to our hypothesis, our results show that only a fixed stimulation protocol leads to a persistent increase in post-stimulation alpha power as compared to sham. Furthermore, in none of the stimulated groups significant modulation of detection performance occurred. While the lack of behavioral effects is inconclusive due to the short selection of different phase bins and trials, the physiological results suggest that a constant stimulation with a fixed frequency is actually beneficial, when the goal is to produce persistent synaptic changes.

## Introduction

Non-invasive methods of brain stimulations like transcranial alternating current stimulation (tACS) find increasing use in neuroscience (Antal et al., 2017; Veniero et al., 2019). tACS is assumed to modulate endogenous brain oscillations in a frequency specific manner. It is frequently used as a tool in intervention studies, with the aim of exploring the functional role of brain oscillations for cognitive processes (Thut et al., 2012; Bergmann et al., 2016; Herrmann et al., 2016). In the past, tACS was successfully used to modulate cognitive functions like visual and auditory perception (Neuling et al., 2012; Brignani et al., 2013; Kasten et al., 2018a), memory (Vosskuhl et al., 2015; Alekseichuk et al., 2016), motor functions (Feurra et al., 2011b), and attention (Kasten et al., 2020). There is also growing research in clinical applications (Clancy et al., 2018; Mellin et al., 2018; Ahn et al., 2019; Alexander et al., 2019). There are currently two presumptions about how tACS achieves its effect. The first is entrainment of ongoing brain oscillations to the driving frequency during stimulation (Thut et al., 2011; Herrmann et al., 2016; Liu et al., 2018). According to the laws of entrainment an oscillator (like a brain rhythm) will synchronize to another coupled oscillator (like tACS), if their frequencies have a close match, or if the driving force of the external oscillator is very high (Pikovsky et al., 2002). The second presumed mechanism is a lasting change of synaptic plasticity of the stimulated networks (Zaehle et al., 2010; Vossen et al., 2015; Liu et al., 2018; Wischnewski et al., 2018). While most effects of tACS occur during the application of the stimulation (online) (Feurra et al., 2011a; Polanía et al., 2012; Brignani et al., 2013), there are also offline-effects that show that functional changes (Marshall et al., 2006; Garside et al., 2014; Kasten and Herrmann, 2017) as well as physiological changes (Reato et al., 2013; Kasten et al., 2016) persist for some time after the end of the stimulation as measured in the electroencephalography (EEG) and magnetic encephalography (MEG).

Recent work has pointed out that the effects of tACS can be quite inconsistent (Veniero et al., 2017; Clayton et al., 2018; Fekete et al., 2018; Sliva et al., 2018), and it has been proposed that the transferability of tACS-findings may be limited by a variety of factors: the dependency of the effects on brain states (Feurra et al., 2013; Herrmann et al., 2013; Neuling et al., 2013; Alagapan et al., 2016), the challenges that come with individual differences in brain anatomy (Krause and Cohen Kadosh, 2014), and the close match between stimulation frequency and brain rhythm that is required according to the laws of entrainment (Fröhlich, 2015; Notbohm et al., 2016). The expected decrease of the stimulation effect by a growing deviation between tACS-frequency and endogenous rhythm was already shown in *in-vitro* and animal studies (Schmidt et al., 2014; Negahbani et al., 2019) and a growing amount of studies suggest a similar role of mismatching frequencies in humans (Vossen et al., 2015; Stecher et al., 2017; Kasten et al., 2019).

A promising approach to address many of the challenges of tACS are so-called “closed-loop” setups. Instead of pre-determining stimulation parameters, from experience and models alone, the parameters are dynamically tuned to the current brain activity in near real time (Boyle and Frohlich, 2013; Wilde et al., 2015; Bergmann et al., 2016; Karabanov et al., 2016; Thut et al., 2017). Respective novel approaches of applying frequency and phase specific tACS corresponding to current brain activity have shown promising results in memory consolidation during sleep (Jones et al., 2018; Ketz et al., 2018) and phase-dependent modulation of the α-rhythm via closed-loop tACS are currently studied (Zarubin et al., 2020).

In this study, we aim to address the problem of frequency specific tACS in the α-range. We employed a closed loop stimulation protocol with adaptive tACS-frequency and tested it against established, fixed tACS-protocols using a single, pre-determined frequency. Previous tACS research in α-band modulation relied on (rapid) preliminary estimation of the individual alpha frequency (IAF) before stimulation (e.g., Zaehle et al., 2010) or even stimulation at a prefixed frequency (Helfrich et al., 2014). The rapid estimation of the IAF before stimulation is usually limited by the scarce amount of data and the quick analysis. Moreover, recent research suggested that the alpha-activity is not as frequency-stable as previously expected (Haegens et al., 2014; Mierau et al., 2017; Benwell et al., 2019). Therefore, a growing amount of studies found a mismatch between the predetermined individual stimulation frequency and the prevalent IAF as established post stimulation by the thorough analysis of more abundant EEG-data (Vossen et al., 2015; Stecher et al., 2017; Stecher and Herrmann, 2018; Kasten et al., 2019). While these studies were not perfectly balanced to explore the effects of the occurring mismatches, their results suggest that a portion of effects of tACS in the α-band are caused by deviation between IAF and ISF. Under the assumption, that entrainment is a necessary perquisite for tACS-effects, such a deviation between driving and endogenous frequency could decrease or prohibit a synchronization of brain rhythms to the tACS. In order to explore whether the effects of tACS can be increased by accounting for shifts in the ongoing α-activity, we designed an experiment where the stimulation frequency was continuously matched to the current prevalent peak-frequency of the α-activity, by adapting a new ISF (individual stimulation frequency) every 8 s from a posterior EEG-recording and stimulating in intermittent epochs of 8 s. This intermediate design is necessary, as the stimulation introduces a substantial artifact into the recording, rendering the analysis of data obtained during tACS extremely difficult (Noury et al., 2016; Herrmann and Strüber, 2017; Kasten et al., 2018b). tACS-protocols employing intermittent 8 s epochs with a cumulative length of 11–15 min were previously shown to be the shortest possible duration to produce physiological aftereffects of increased band-power (Vossen et al., 2015), while shorter epochs such as 1 and 3 s showed no effect (Strüber et al., 2015; Vossen et al., 2015). To compare the effect of the adaptive stimulation to the conventional fixed stimulation, we contrasted the results to a sham-stimulation and a fixed-frequency condition. To maintain a consistent state of mental alertness, we coupled the stimulation to a visual detection task, where changes in luminance, phase-locked to specific cycles of the stimulation, had to be detected.

We hypothesized that both verum tACS-groups would show an increased α-power after stimulation when compared to the sham group. Furthermore, we expected a larger increase of power following the adaptive tACS (closed-loop) when compared to the stimulation at a predetermined fixed frequency, as a closer match of the stimulation frequency to the endogenous alpha activity should result in a higher proportion of entrainment during the stimulation. This higher proportion of entrainment should be accompanied by a stronger effect of synaptic plasticity in the underlying neuronal networks. We further expect a larger modulation of the detection performance within the stimulated epochs in the closed-loop condition as the applied tACS-waveform will better coincide with peaks and troughs of the ongoing α-activity, thereby increasing and decreasing the chance of visual detection in the respective phases (Mathewson et al., 2009).

## Materials and Methods

### Participants

Sixty students of the University of Oldenburg aged between 18 and 35 (mean: 24.4 ± 3 years) participated in the study. Each gave written informed consent to participate and have their results anonymously published and received monetary compensation. The participants were subdivided into three groups: sham, fixed stimulation frequency (fIAF), and closed-loop stimulation (cIAF). The groups were counterbalanced for gender. Group assignment was done randomly by a computer after subject preparation and information. Due to equipment failure six participants were omitted from further analysis. Additionally, two participants of the sham-group showed an abnormal increase in α-activity (5 σ outside of the population mean) and were discarded from the statistics. The resulting group sizes were sham *N* = 17 (7 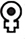), cIAF *N* = 17 (9 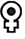), and fIAF *N* = 18 (8 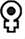).

The participants were informed about the general goal and the procedure of the experiment and filled out a short questionnaire regarding the exclusion criteria. All participants reported to be free of psychiatric medication at the time of the experiment. Subjects stated no history of epilepsy, no neurological or psychiatric disorders, no cognitive impairments, no intracranial metal or cochlear implants, and normal or corrected to normal eyesight. After finishing the experiment participants were asked whether they thought they were stimulated and to complete a short questionnaire assessing possible adverse effects of tACS (Brunoni et al., 2011). All participants were naïve regarding the aim of the study. The study was approved by the Commission for Research Impact Assessment and Ethics at the University of Oldenburg.

### Experimental Setup

**FIGURE 1:**
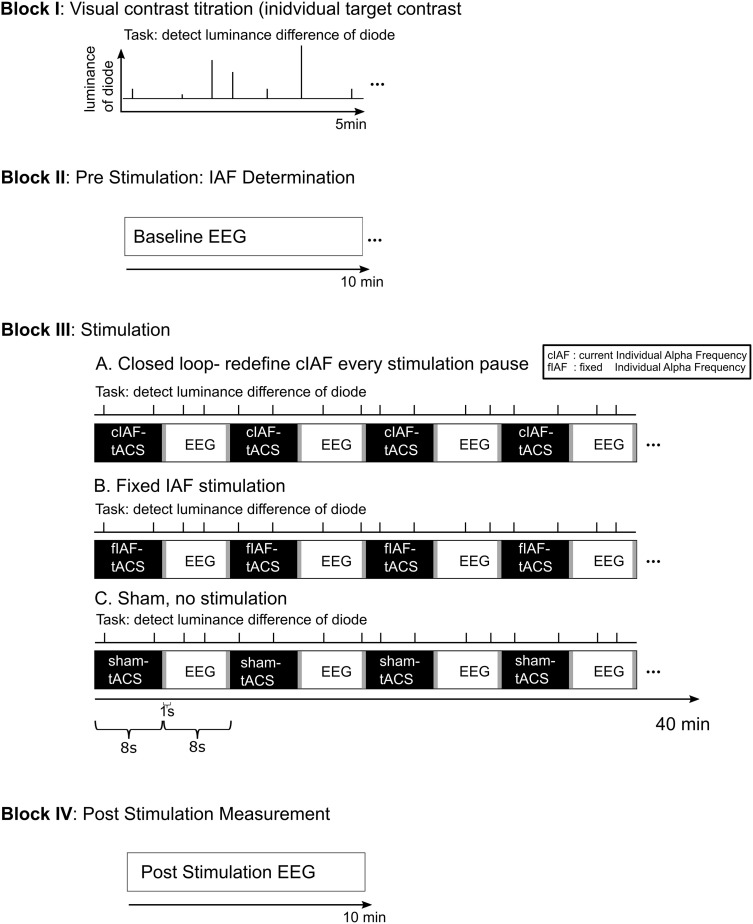
Experimental Procedure. The experiment was divided into four blocks: 1. The participants performed a 5 min titration procedure to establish an individual luminance threshold for the detection task. 2. A 10 min EEG recording was conducted pre-stimulation. From this data, the individual stimulation frequency for the fixed stimulation and the sham group was established. 3. The stimulation part consisted of 150 epochs of 8 s of stimulation interleaved with 8 s stimulation free EEG-recording. For the fixed stimulation and the sham group, the predetermined ISF was used. For the closed loop group, the stimulation frequency for each epoch of stimulation was determined from the preceding stimulation-free epoch. During the whole stimulation block, the participants performed a visual luminance detection task. 4. The session concluded with a further 10 min of stimulation free EEG.

Participants were seated in a dimly lit room in front of a light emitting diode (LED) in 50 cm distance centered between their eyes. The experimental setup is depicted in Figure 1: Following the preparation of the electrodes, participants performed a staircase procedure to determine the individual brightness threshold for the detection task. The one up/one-down staircase started at a photodiode voltage of 2.365 V and decreased/increased by 0.001 V for every correct/incorrect response, until 15 reversals were reached. The individual detection threshold was then calculated as the mean voltage of the reversals 5–10. During a 1-min EEG recording, an individual eye-blink threshold was determined. The subsequent experimental session started with a 10 min pre-stimulation EEG, followed by a 40 min part during which intermittent tACS was administered. The stimulation part was followed by another 10 min EEG recording. During the whole session, participants performed a visual detection task. The stimulation block was subdivided into 150 epochs containing 8 s of stimulation and 8 s of interleaved EEG recording. In the closed-loop and the fix-frequency groups, tACS was applied for 8 s in each sequence (including 1 s of linear fade in). The sham stimulation consisted of a 1 s fade in followed by a 1 s fade out at a fixed frequency. This application of a current every 8 s in all three conditions should ensure a better blinding than established methods of only comparatively very short placebo-conditions, which have been recently criticized (Turner et al., 2021).

The participants were tasked to detect changes in the LED’s brightness and react by pressing a button with their right index finger. The changes in brightness lasted 10 ms and were a reduction in LED-voltage by the previously determined individual threshold. The changes in brightness are referred to as targets in the following. Targets occurred at four phase positions relative to the applied sinusoidal tACS: at 0°, 90°, 180°, or 270°. Two targets were presented per stimulation sequence (8 s) and then likewise presented at the same positions of the subsequent interleaved EEG-sequence. Targets appeared after the stimulation fade-in of 1 s and were jittered by ±1.75 s in the first and second half of the stimulation sequence. The order of the tACS phase angle at the time of the target presentation was randomized between subjects.

### EEG and Individual Alpha Frequency Estimation

The EEG was measured with 25 sintered Ag-AgCl electrodes fitted in an elastic cap (EasyCap, Falk Minow, Munich, Germany). A standard 10–20 layout was applied with a vertical EOG-electrode, referenced to the tip of the nose. The ground electrode was positioned at FPz. Impedances were kept under 10 kΩ. The signals were recorded via BrainVision Recorder (BrainProducts GmbH, Gilching, Germany) with a resolution of 16.35 nV and at a sampling rate of 250 Hz, to favor faster processing in the closed loop stimulation. A high cutoff filter of 250 Hz and a low cutoff filter at 0.1 Hz were applied during the recording.

**FIGURE 2:**
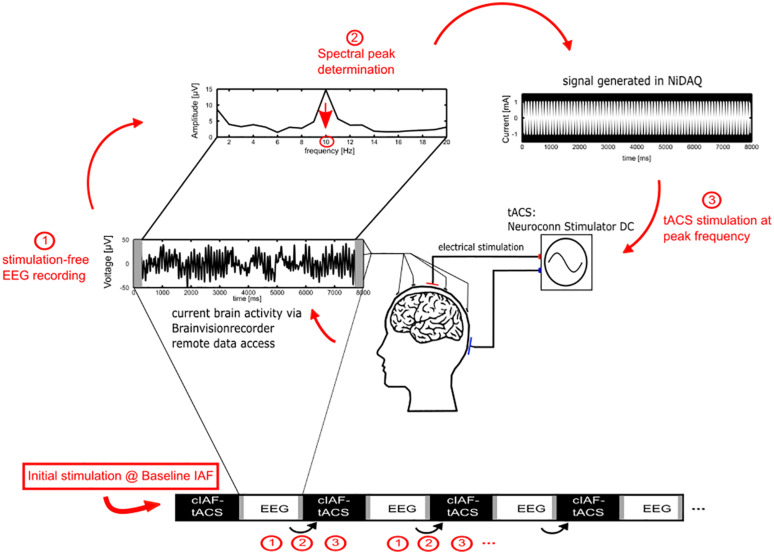
Adaptive IAF determination for the closed-loop group. 1. During the stimulation part, the current individual stimulation frequency for the closed-loop group is determined by Fourier transforming the EEG data of the preceding EEG epoch of 7 s (omitting 1 s to avoid edge effects of the stimulation epochs). 2. The peak of the power spectrum within the alpha range, corrected for the 1/f characteristic, is chosen as the new stimulation frequency. 3. A new stimulation signal is generated using a National Instruments digital to analog converter that streams the stimulation data to a NeuroConn stimulator for the next epoch of tACS.

In order to determine the initial individual peak alpha frequency for the stimulation, the 10 min pre-stimulation EEG recording was used. For the fixed-frequency and the sham group, the estimated peak frequency was used as the ISF for the remainder of the experiment. For the closed-loop group, a new ISF was determined from 7 s of each interleaved EEG-sequence (see Figure 2). For the estimation of the frequency, the data of electrode Pz was subdivided into 1 s sequences, zero padded to 1250 sampling points to offer a resolution of 0.2 Hz and multiplied with a Hanning-window. Data-seconds containing values above the individual eye blink threshold were discarded. To correct for the 1/f characteristic of the power spectrum, the power at each frequency was multiplied with the respective frequency. The IAF was determined as the maximum value in the power spectrum between 7.2 and 12.8 Hz. In order to ensure that the value reflected an actual peak in the spectrum rather than noise, an additional constraint was applied, requiring that the power at the identified maximum was larger than the average power in the whole band (the mean of 7.2 and 12.8 Hz) plus one standard error. If no IAF could be found, a stimulation of 10 Hz was applied.

### Electrical Stimulation

For tACS, two surface conductive-rubber electrodes (5 × 7 cm) were centered at Cz and Oz underneath the EEG recording cap. The electrode’s positions were chosen in order to stimulate the parieto-occipital cortex, in accordance to previous studies (Neuling et al., 2013). The rubber electrodes were fixed to the head using Neurodiagnostic Electrode Paste (Ten20; weaver and company) and impedance was kept below 10 kΩ. A stimulation current of 1 mA (peak to peak) was applied according to the group at the individual stimulation frequency with a battery-operated NeuroConn Stimulator DC (Neurocare, Illmenau, Germany). The stimulation was only exerted during the 40 min stimulation part in 8 s sequences, resulting in a total of 20 min of stimulation. The stimulation signal was continuously controlled within a MATLAB loop, by accessing the BrainVision-Recorders remote data access port, establishing the ISF by the procedure as described above and generating a sinusoidal signal with the respective parameters at 1,000 Hz sampling frequency. The generated signal was streamed via a digital-to-analog converter (DAQ NI USB 6229, National Instruments, Austin, TX, United States) to the remote port of the stimulator [Figure 2(3)].

### Post-measurement EEG Data Analysis

The EEG data were analyzed using MATLAB 2018a and the fieldtrip toolbox (Oostenveld et al., 2011). The stimulation epochs were cut from the data, and linear trends and the mean were subtracted from each channel. The data were then filtered using a 1 Hz high pass filter and a 100 Hz low pass filter, using a two-pass Butterworth filter of sixth order. In order to clear the data from raw muscle and movement artifacts, trials containing voltage deflections exceeding > 150μV were discarded. The remaining trials were fed into an Independent Component Analysis (ICA) and eye-movement components manually selected and rejected. The data was then rearranged into a 10 min pre-stimulation block, 149 intermittent 7 s epochs between stimulation epochs (the last block was omitted due to a strong electrical artifact caused by the NiDAQ-shutdown), and a 10 min post-stimulation block. Blocks were subsequently divided into 1 s trials and Fourier transformed, using a 5 s zero padding and a Hanning-taper. Alpha peak power in each block was determined by identifying the peak α-power (maximum between 6.5 and 13 Hz) at electrode Pz in the averaged spectrum of each block.

### Statistical Analysis

Statistical analysis was performed using R 4.0.2 (R Foundation for statistical Computing, Vienna, Austria). The behavioral analysis was conducted on the detection performance data during the stimulation measurement. In order to explore phase-dependent modulatory effects on the visual detection task, we calculated the detection performance for the four phase bins during tACS and the four bins in between stimulation epochs for every participant. As we assumed any behavioral modulation to be sinusoidal, we subsequently performed a sine-fit through the four points of performance values for every participant and condition (during tACS, during break) with a fixed frequency of 1 cycle and free values for intercept and amplitude. As the individual latency between visual processing of the stimuli and the tACS field was unknown, we also allowed a random value for phase. For every participant we took the values of the fitted sine during stimulation and the fitted sine during the break and calculated relative values for amplitude and ordinary R^2^ of the fits. We then used a Kruskal Wallis test to check if the behavioral modulation between both conditions differed by group. The hypothesized effect of tACS on post-stimulation alpha power was tested by employing a Kruskal Wallis test on the relative increase in peak power between the groups. This test was chosen as peak power was not normally distributed and did not fulfill the criteria for an ANOVA. As there is no alternative for a non-parametric repeated measures ANOVA, the percent change on peak-power relative to the pre-stimulation measurement was calculated. We also tested the average α-power during the non-stimulated epochs within the stimulation-measurement relative to the pre-stimulation power with a Kruskal Wallis test, to determine if physiological differences were already present during the stimulation part.

## Results

### Adverse Effects

A Kruskal-Wallis test on the reported adverse effects of the stimulation did not reveal any difference in responses between the three groups (all *p* > 0.05). The most frequent reported effects (scores of three or higher) were “trouble concentrating” (*N* = 16) and “tiredness” (*N* = 6). There was also no difference in the believe to have received stimulation (*p* = 0.547), indicating that the blinding worked successfully.

### Behavioral Results

**FIGURE 3:**
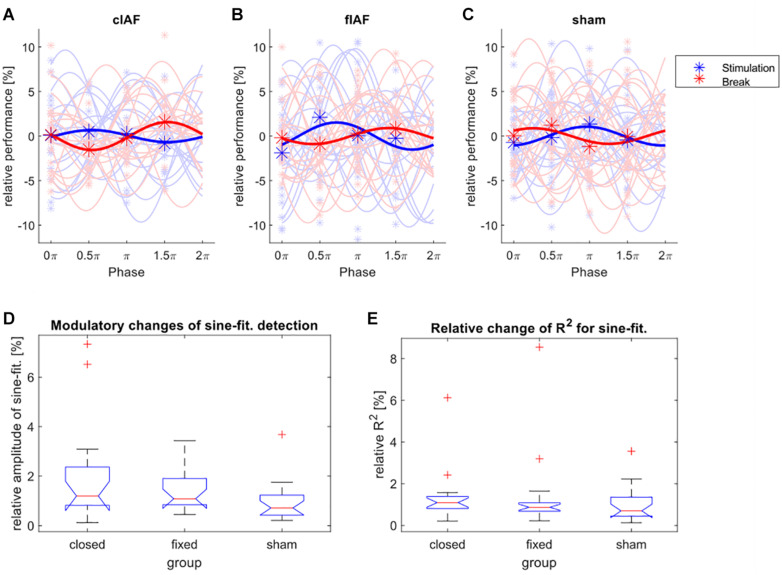
Modulation of detection performance. **(A–C)** Detection performance during the four phase bins (asterisks) and fitted individual sine waves for every participant (light colors) and the average over all participants (dark colors), during stimulation epochs (blue) and between the stimulation epochs (red). The performance is shown relative to the mean over all four phases. **(D)** Boxplot of relative change in amplitude (performance during stimulation divided by performance during break) of the sine-wave fitted on the detection performances of the four phase bins. **(E)** Boxplot of relative changes (stimulation divided by break) of the ordinary R^2^-values of the fitted sine-waves for all three groups.

The detection performance for the targets distributed over the four different phase bins and the fitted sine-waves show no striking differences between groups and conditions (see Figures 3A–C). We tested the relative differences between the amplitudes of the fitted sines during break-trials and stimulation-trials for every group by using a Kruskal Wallis test (see Figure 3D), with the between factor group (sham, cIAF, fIAF). There were no significant differences (*X*^2^ = 4.45, *df* = 2, *p* < 0.108). The same analysis was repeated for the ordinary R^2^ of the fitted sinusoidal (Figure 3E) to explore if the groups differed in how well the modulation of detection was explained by a sine-function. The analysis again revealed no significant differences between the groups (*X*^2^ = 1.98, *df* = 2, *p* < 0.372).

In a recent article, Zoefel et al. (2019) compared different methods for the exploration of phase-dependent modulations of perception. They could show that simple sinus-fit method as we employed it here is not optimal for datasets with a limited number of phase bins and a small number of trials. The most optimal method they tested was a logistical regression with circular predictors. By employing their provided scripts for our dataset, we repeated the behavioral analysis with the described LOG REGRESS FISHER and LOG REGRESS PERM methods. For the LOG REGRESS FISHER-method, the phase of each trial was sine and cosine transformed to obtain a linear predictor. The dichotomous responses of each trial were then included in a regression model. For every participant, two regression models were created: one from trials during stimulation breaks and the second from trials during stimulation. Each regression model was then compared to an intercept-only model by using an *F*-Test. The resulting *p*-values for every participant and condition were then combined according to group using Fisher’s method. For no group or condition the regression model provided a better fit than the intercept only model (all *p* > 0.1). For the LOG REGRESS PERM-method the trials and circular predictors were used to fit a multinomial logistic regression and the resulting root-mean square of the regression coefficients (sine and cosine) was stored for every participant and condition. This process was then repeated 100 times for every participant and condition with randomly permutated phases for all trials, resulting in 100 randomized surrogate datasets for every participant and condition. The average root-mean square of every condition was then compared against the average respective surrogate distributions for every group. The *z*-test was not significant for any group or condition (all *p* > 0.01).

### Physiological Results

**FIGURE 4:**
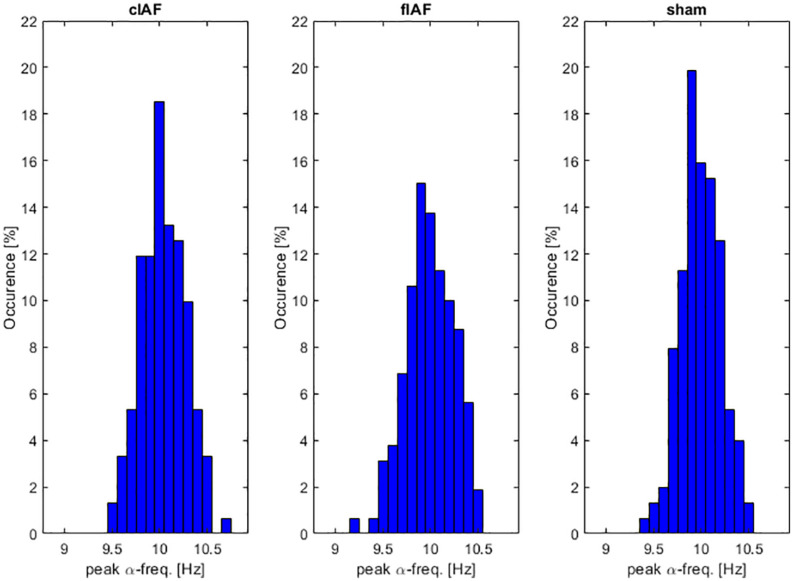
α-frequency distribution. Occurrences of peak α-frequency between stimulation epochs. Histograms show the prevalence of peak-frequencies within the alpha range, averaged over participants.

**FIGURE 5:**
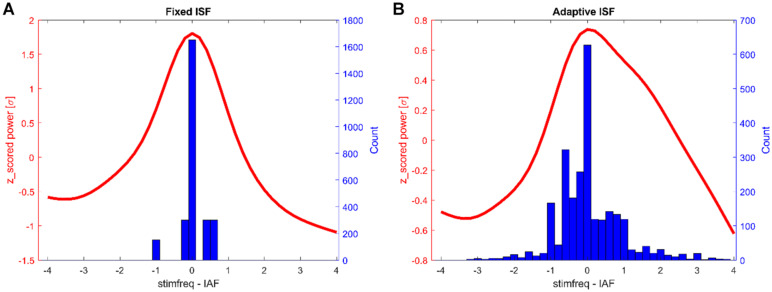
Histograms of tACS-frequencies during stimulation epochs (blue) and the average normalized α-spectrum post stimulation (red, all frequencies centered on post-stimulation α-peak). Shown are the counts for all participants (150 trials each). **(A)** Fixed stimulation group. **(B)** Closed-loop stimulation group.

We first wanted to explore how much the IAF shifted over time and explore whether the shifts differed between the three different groups. As can be seen in Figure 4, all groups showed a variance in peak frequency over all intermediate windows between the stimulation epochs. We tested the number of shifts in frequency by testing the variance in peak frequencies per participant between groups. An ANOVA revealed no significant differences between the frequency-variance between groups [*F*_(__1,49__)_ = 0.4, *p* = 0.645]. As can be seen in Figure 5, the stimulation frequency in both stimulated groups did not always match perfectly with the prevalent IAF as determined from the post-stimulation block. During 19 (σ 11.1) epochs on average per participant in the closed-loop stimulation group, the closed loop system failed to detect an IAF-peak and a stimulation of 10 Hz was applied.

**FIGURE 6:**
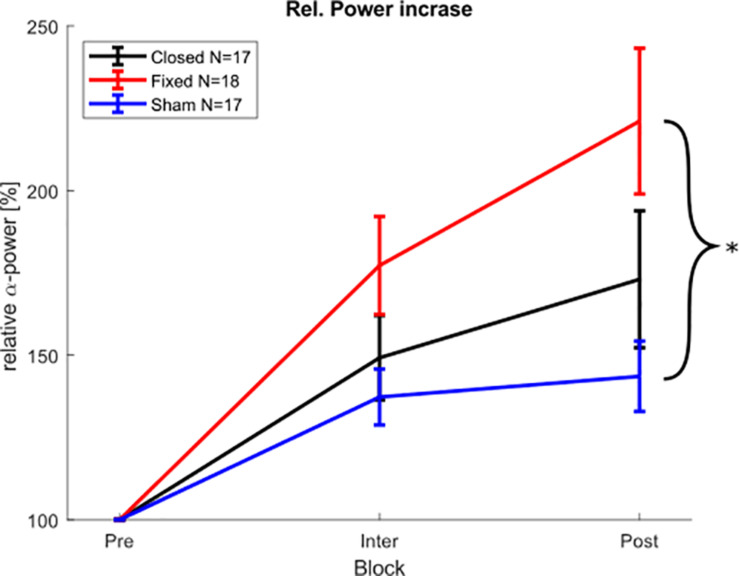
Physiological aftereffect. Average posterior α-peak power during and after stimulation block, relative to pre-stimulation power. Error bars depict the standard error of the mean. The asterisk marks significant differences. The power during the stimulation block was calculated from the 150 stimulation free epochs between the stimulation epochs.

For the physiological results in the post-stimulation block, a Shapiro Wilk test showed that the relative α-power values were not normally distributed in all groups. Therefore, a Kruskal Wallis test was chosen as a non-parametric alternative to an ANOVA. The Kruskal Wallis test showed a significant difference of α-power changes between the groups (*X*^2^ = 6.8979, *p* < 0.032), and a pairwise Wilcoxon rank sum test (Bonferroni-Holm corrected) revealed that the fixed-stimulation group showed significantly increased power as compared to the sham group (*p* < 0.025) (see Figure 6), whereas the comparison between closed loop- group and sham group was not significant (*p* < 0.474). The Kruskal Wallis test on the α-power (averaged over all stimulation-free epochs) during the stimulation part revealed no such differences between the groups (*X*^2^ = 3.5283, *p* = 0.171).

**FIGURE 7:**
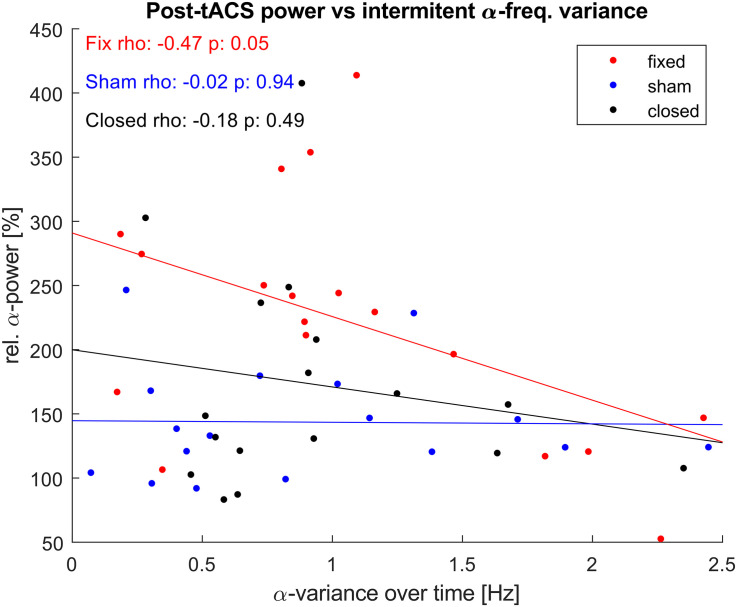
α-power vs. α-frequency variance. Correlation between relative posterior α-power post stimulation and the variance of the IAF displayed during the intermediate unstimulated-epochs. One dot represents power and α-variance values of a single participant. Lines show the least-square error lines per group.

As the aftereffect of α-tACS is known to depend on match between stimulation frequency and the current IAF, we explored if the observed power-increase correlated with the variance that the IAF showed during the unstimulated epochs (Figure 7). While both stimulated groups showed a negative correlation between individual variance in IAF over time, this correlation was only significant for the fIAF-group.

**FIGURE 8:**
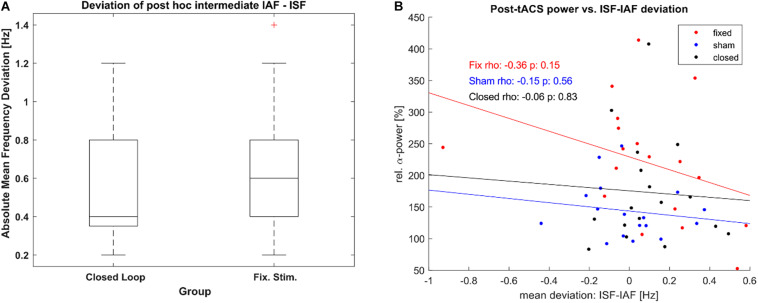
Stimulation accuracy. **(A)** Boxplot of Frequency Deviation [mean absolute difference between estimated stimulation frequency (ISF) of each epoch and the determined α-frequency (IAF) as per *post-hoc* analysis] for both the closed loop tACS and the fixed frequency tACS group. Boxes mark the ends of the 25th and the 75th percentile of the samples’ distributions, the horizontal lines mark the median of each group. The whiskers correspond to +/–2.7σ of the data. **(B)** Correlation between relative posterior α-power post stimulation and the mean deviation between ISF and IAF during the intermediate unstimulated-epochs. One dot represents power and frequency-deviation values of a single participant. Lines show the least-square error lines per group.

Furthermore, as the adaptive frequency estimation was based on a quick and rough method, we explored the resulting accuracy of both stimulated conditions as defined by the difference between ISF and IAF per epoch as established with *post-hoc*. We did so in order to establish that the difference in post-stimulation α-power between both groups was not based on a lack of stimulations accuracy within the closed-loop group. Both stimulated groups did not show a significant difference mean deviation between ISF and IAF (see Figure 8A), as tested by a Wilcoxon rank sum test (Z = −0.59, *p* = 0.56), maintaining that our results were not caused by a lack of stimulation accuracy in the closed-loop condition. Additionally, we tested whether the post-stimulation power was dependent on the accuracy of stimulation but we could not find significant correlation for any group (see Figure 8B).

## Discussion

Our aim in this study was to study the effectiveness of a closed loop α-tACS system where the stimulation frequency is continuously adapted to the current endogenous alpha-frequency. We found that stimulation with a fixed frequency led to an increase of post-stimulation α-power when compared to sham. This increase was not significant during the stimulation-measurement when we analyzed the unstimulated epochs. The post-stimulation increase is comparable to previous findings (Neuling et al., 2013; Vossen et al., 2015; Kasten et al., 2016). Surprisingly, we could not find evidence for the hypothesized stronger increase in post stimulation α-power when we constantly adapted the stimulation frequency to the current individual α-frequency.

Additionally, the tACS did not led to difference in phase-dependent modulation of visual detection between the stimulated and the unstimulated epochs. This effect was also absent when we employed a more sensitive method suggested by Zoefel et al. (2019). Previous attempts to modulate visual perception in a phase-specific way by brain stimulation have shown mixed results (Kasten and Herrmann, 2020). Evidence for phasic modulation has been shown in a visual-oddball task using tACS (Helfrich et al., 2014) and in a discrimination task using rTMS (Jaegle and Ro, 2014). However, more recent studies with similar detection tasks as employed here failed to find phase-specific effects for tACS (de Graaf et al., 2020) and oscillating transcranial current stimulation (otCS) (Sheldon and Mathewson, 2018). The differences in the parameters of task, stimulation, and analysis makes a direct comparison quite difficult. Different approaches have been used to uncover effects of phasic modulation in the past. Only recently, a comprehensive comparison of different approaches and a recommendation for a common procedure has been proposed by Zoefel et al. (2019). Their results suggest that a number of trials exceeding those used in our and others’ studies are necessary to robustly uncover effects of phasic modulation on behavior.

This suggests that perhaps the choice of our behavioral task itself was suboptimal for the exploration of the question whether an adaptive stimulation frequency is beneficial in functional modulation over a fixed frequency approach. The physiological outcomes of our study, however, suggest that adherence to a fixed stimulation frequency can be beneficial if the goal of the stimulation is to produce a robust aftereffect.

Previous studies found a dependence of the post-stimulation power on the mismatch between ISF and IAF (Stecher et al., 2017; Stecher and Herrmann, 2018; Kasten et al., 2019), while some work even suggests that a stimulation frequency slightly below the IAF yields stronger plasticity effects (Herrmann et al., 2013; Vossen et al., 2015). The prevalent notion suggested that a closer fit between stimulation frequency and endogenous frequency would lead to an increased amount of entrainment during which the synapses of the underlying oscillatory networks are strengthened. This notion is supported by findings that link the aftereffects of tACS to NMDA-receptors (Wischnewski et al., 2018). One possible explanation as to why this aftereffect only occurs for fixed ISFs and not for adaptive ISFs is that during a fixed-ISF stimulation, those networks with a fitting resonant frequency experience synaptic strengthening according to the rules of spike timing dependent plasticity (STDP) (Song et al., 2000; Feldman, 2012) while an ever-shifting ISF will cause conflicting effects in networks of neighboring frequencies. Previous modelling studies suggest that tACS shifts the probability of spikes occurring within a network in a phase-dependent way (Ozen et al., 2010; Reato et al., 2010). Within a recurrent network of fitting eigenfrequency pre-synaptic spikes occur more likely within a time-window, that is “causal” for post-synaptic spikes (Herrmann et al., 2013), thereby leading to long term potentiation (LTP) over the course of multiple tACS-cycles due to NMDA-receptor mediated plasticity. If the tACS-frequency shifts into a region where spikes are occurring outside of this time-window, either no plasticity effects may occur or the probability of spikes occurring may even be shifted to time-windows where the spikes occur after post-synaptic activity, now causing synaptic depression in networks that were strengthened in the previous stimulation epoch. This would suggest that within our closed-loop stimulation group, the size of the tACS-aftereffect should depend on the stability of the ISF. While the results of the fixed stimulation group hint into this direction (c.f. Figure 5), the wide array of parameters on which such a stability depends (positive and negative frequency shifts, sequence of frequencies, number of failed IAF-estimations and prevalence of different frequencies) make it hard to find a single suitable testable predictor for the closed-loop stimulation.

The universality of our results is mainly limited by three design-choices: First, the setting of 8 s epochs of stimulation was motivated by previous results, showing that intermittent tACS of 8 s show comparable effect to continuous stimulation and the offered opportunity to perform rough artifact-correction methods. The method, however, neglects any variance of the peak IAF over the respective time. It is possible that the prevalent α-frequency encountered a shift within 8 s, resulting in an unfitting stimulation frequency for the following epoch and offering only a slow adaptation to changes. Intermittent tACS-protocols that employ substantially shorter or longer stimulation epochs might yield different physiological and functional results, with very-short stimulation epochs or even a true “online” approach offering the opportunity of instant-frequency adaption, omitting larger jumps in stimulation frequency. Second, the fast procedure we employed to quickly estimate the IAF during the stimulation block could result in an insufficient stimulation accuracy due to lacking robustness against stronger artifacts and the reliance on zero padded 1 s chunks. This seems evident in the fact that the deviation between stimulation frequency and *post-hoc* established IAF, while smaller, was not significantly better in the adaptive condition compared to the fixed-frequency condition. Future closed-loop designs could improve the frequency-estimation by relying on online artifact techniques developed for Brain Computer Interfaces (Schlögl et al., 2007) and methods to compute the instantaneous frequency (Cohen, 2014). On a minor point, the choice to stimulate at a fixed frequency of 10 Hz in our adaptive design instead of reusing the last estimated IAF might have been less than ideal. Such sudden shift could cause a ISF that is too far from the endogenous frequency to have any effect. Given that the IAF will probably not change as drastically within this time-window, it might have been better to just repeat the last employed ISF. Third, our behavioral detection task consisted of visual stimuli presented at only four different phase bins with only 75 trials per phase. This number is rather low and would require a large effect size to statistically uncover phasic modulations as could be shown by Zoefel et al. (2019). Their findings suggest that a maximization of the number of trials per phase bin should be sought for in future studies in order to uncover effects of phase-dependent modulation.

In this study we successfully employed an intermittent closed loop stimulation setup. While we found no evidence for our originally hypothesized advantages of such a system over a fixed stimulation setup for the evocation of physiological changes and functional modulation of brain rhythms, we could demonstrate that a fixed stimulation setup produces more robust physiological aftereffects. We could, however, not show that the physiological aftereffects were in any way associated with perceptual changes. The absence of any behavioral effects in the fixed-frequency stimulation group compared to sham likely means that our paradigm was not satisfactorily designed to show any advantages of an adaptive closed loop stimulation protocol. Futures studies should employ behavioral tasks where phasic modulation by tACS has been successfully shown before in order to properly address this research question.

## Data Availability Statement

The raw data supporting the conclusions of this article will be made available by the authors, without undue reservation.

## Ethics Statement

The studies involving human participants were reviewed and approved by the Commission for Research Impact Assessment and Ethics at the University of Oldenburg. The patients/participants provided their written informed consent to participate in this study.

## Author Contributions

AN, FK, and CH: designed the study. AN and FK: acquired the data. HS, AN, and FK: analyzed the data. HS, AN, FK, and CH: wrote the manuscript. All authors contributed to the article and approved the submitted version.

## Conflict of Interest

CH has filed a patent application on brain stimulation and received honoraria as editor from Elsevier Publishers, Amsterdam. The remaining authors declare that the research was conducted in the absence of any commercial or financial relationships that could be construed as a potential conflict of interest.
